# Preparation and Enzyme Degradability of Spherical and Water-Absorbent Gels from Sodium Carboxymethyl Cellulose

**DOI:** 10.3390/gels8050321

**Published:** 2022-05-20

**Authors:** Sayaka Fujita, Toshiaki Tazawa, Hiroyuki Kono

**Affiliations:** 1 Division of Applied Chemistry and Biochemistry, National Institute of Technology, Tomakomai College, Tomakomai 059-1275, Japan; 2 R&D Center, S.T. Corporation, Shinjuku-ku, Tokyo 161-0033, Japan; t-tazawa@st-c.co.jp

**Keywords:** carboxymethyl cellulose, superabsorbent gels, enzymatic biodegradability, spherical control, molding processability

## Abstract

To synthesize a biodegradable alternative to spherical polyacrylic acid absorbent resin, spherical hydrogel particles were prepared from carboxymethyl cellulose (CMC) dissolved in an aqueous solution, using ethylene glycol diglycidyl ether (EGDE) as a crosslinking agent. The effect of varying the initial CMC concentration and feed amount of EGDE on the shape, water absorbency, water-holding capacity, and enzyme degradability of the resultant CMC hydrogels was determined. The reaction solution was poured into fluid paraffin, and spherical hydrogel particles were obtained via the shear force from stirring. The shape and diameter of the spherical hydrogel particles in the swollen state depended on the CMC concentration. The spherical hydrogel particles obtained by increasing the amount of EGDE resulted in a decrease in absorbency. Additionally, all the spherical hydrogel particles were degraded by cellulase. Thus, spherical biodegradable hydrogel particles were prepared from CMC, and the particle size and water absorption of the hydrogel could be controlled in the range of 5–18 mm and 30–90 g·g^−1^ in the swollen state, respectively. As an alternative to conventional superabsorbent polymers, the spherical CMC hydrogels are likely to be useful in industrial and agricultural applications.

## 1. Introduction

Superabsorbent polymers (SAPs) can absorb and retain extremely large amounts of water [1]. The most popular SAP is crosslinked sodium polyacrylate (SPA), which is industrially produced by copolymerizing acrylic acid and acrylic acid sodium salt [2]. SAPs are widely used in various applications, such as baby diapers, feminine sanitary products, adult incontinence pads, carriers for air fresheners, and agricultural water retention materials. One of the biggest drawbacks of industrial SAPs, including SPA, is that they are non-biodegradable, despite their widespread use in disposable goods [3]. As society moves toward more sustainable and fossil fuel-free commodities and processes, developing biodegradable SAPs is imperative to prevent microplastic accumulation associated with synthetic polymers that causes environmental pollution.

The primary candidates of raw materials for biodegradable SAPs are naturally occurring and inedible polysaccharides, such as cellulose [4], chitin [5], chitosan [6], guar gum [7], starch [8], and carrageenan [9]. These polysaccharides exhibit biocompatible and biodegradable properties; thus, they are of considerable interest for the development of environmentally friendly and biocompatible materials. Structurally, these polysaccharides are polymers consisting of neutral and/or glycosidically bonded amino sugars; hence, they exhibit no water-absorbing properties. Therefore, various methods have been reported to impart the same water-absorbing performance as that of SAPs onto these polysaccharides, while maintaining their biodegradability [4,5,6,7,8,9]. Most of these methods are based on the substitution of the hydroxyl and/or amino groups of the polysaccharide chain with sodium carboxylate groups and intermolecular crosslinking between the polysaccharide chains. For example, carboxymethyl cellulose sodium salt (CMC), a water-soluble cellulose derivative, can be converted into a biodegradable superabsorbent polymer by intermolecular crosslinking using diepoxy crosslinking agents, such as ethylene glycol diglycidyl ether (EGDE). Here, the sodium carboxylate groups of the CMC and the intermolecular crosslinking between the CMC chains are responsible for the absorption and retention of water molecules inside the structure, respectively [10,11,12]. The same method was used to develop water-absorbent polysaccharides. The biodegradability of these polysaccharide-based water-absorbent polymers strongly depends on the crosslinking agent used for their preparation and the crosslinking density of the resultant polymer; an increase in the crosslinking density generally leads to a decrease in biodegradability. Therefore, when designing a polysaccharide-based SAP, it is important to consider not only its water-absorbing properties but also the crosslinking agent and crosslinking density.

Difficulty in molding processability is another challenge to overcome during the synthesis of polysaccharide-based SAPs. SAPs with different shapes are utilized for different purposes. The powder form of SAP is used for baby diapers and feminine sanitary products, while the spherical form is used as a carrier for air fresheners and horticultural water retention materials. Most polysaccharide-based SAPs reported to date are obtained as solid lumps, which are then crushed into a powder [13,14]. CMC-based SAPs are no exception. The majority of the CMC-based SAPs are obtained in irregular shapes. This is because polysaccharide-based SAPs, including CMC-based SAPs, are not suitable for injection molding or extrusion molding, which are both molding processes for resins, due to their poor thermal plasticity and solubility in organic solvents. In the case of SPA, the spherical shape of the resin can be controlled by emulsifying droplets of the starting material, consisting of a mixture containing acrylic acid, acrylic acid sodium salt, crosslinking agent, etc., in a poor solvent. In addition, the spherical resin size can be regulated by controlling the emulsified micelle size through the selection of surfactants [15,16]. Therefore, spherical resin particles of various sizes are available for application, while particles with a diameter of 5–15 mm in the swollen state are mainly used for disposable applications. On the other hand, research on the synthesis of polysaccharide-based spherical particles has primarily focused on nanoscale particles for biological applications, such as drug delivery carriers [17,18]. This method forms micelles from a polysaccharide aqueous solution in a poor solvent using a surfactant, followed by producing nanomicelles by ultrasound irradiation [19,20,21]. This makes it difficult to synthesize polysaccharide-based SAPs for general applications. Therefore, to develop polysaccharide-based SAPs as alternatives to SPA, it is necessary to develop synthesis technologies that allow for the precise control of their shape and size in general and disposable applications.

**Figure 1:**
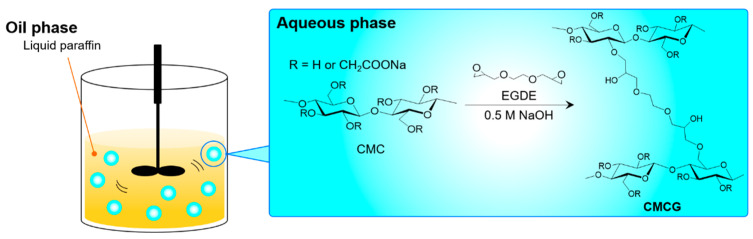
Preparation of the spherical carboxymethyl cellulose sodium salt hydrogel (CMCG) by the formation of a water droplet in liquid paraffin.

In this study, a polysaccharide-based SAP with a water absorption capacity and shape comparable to that of the existing spherical SAP was prepared using CMC and EGDE as the crosslinking agent. An aqueous alkaline solution, comprising CMC and a crosslinking agent, was added to liquid paraffin, and the shear force of stirring the liquid paraffin and the surface tension of the water–oil interface produced spherical particles of the alkaline solution (Figure 1). The initial CMC ratio and the feed amount of EGDE were varied to determine their effects on the shape, water absorbency, water-holding capacity, and cellulase degradability of the resultant CMC hydrogel (CMCG). Commercial SPA particles were used as a comparative reference. This study addressed the challenge of molding processability of polysaccharide-based SAPs and contributed to the development of a sustainable society through the use of biodegradable carriers for air fresheners and horticultural water retention materials.

## 2. Results and Discussion

### 2.1. Preparation and Characterization of the Spherical CMCG

#### 2.1.1. Optimization of the Initial CMC Concentration

To obtain spherical CMCG particles, the reaction mixture containing CMC and EGDE dissolved in the alkaline solution was dropped into liquid paraffin and stirred to facilitate the crosslinking reaction (Figure 1). After being placed in the liquid paraffin, the CMC mixture precipitated at the bottom of the beaker at a stirring speed of 200 rpm. Thus, these samples were lumpy, with no spherical particles formed. Because the surfacing of the reaction mixture prevented adhesion to the stirring blade and the bottom of the beaker, spherical particles were obtained at stirring speeds exceeding 300 rpm. The particle size was affected by the stirring speed, and the particle size dropped as the stirring speed increased (Figure S1). The shear force increased as the stirring speed increased. The increased stirring speed was expected to increase shear force and slice the CMC mixture into smaller droplets [22,23], reducing the CMCG particle size. While maintaining a constant stirring speed of 300 rpm, a series of seven CMCG samples were prepared by varying the CMC concentration and EGDE feed amount (Table 1) to investigate their effects on the shape, water absorption properties, and biodegradability of the CMCGs obtained.

To investigate the effect of the CMC concentration on the formation of spherical shapes, **CMCG_2.5,0.4_**, **CMCG_5,0.4_**, **CMCG_7.5,0.4_**, **CMCG_10,0.4_**, and **CMCG_15,0.4_** were prepared by setting the initial CMC concentrations to 2.5, 5.0, 7.5, 10, and 15 wt%, respectively (Table 1). The feed mass ratio of EGDE to CMC (EGDE/CMC) was fixed at 0.4. The yields of the spherical CMCG are summarized in Table 1. For **CMCG_2.5,0.4_** and **CMCG_5,0.4_**, when the reaction mixture was placed in the liquid paraffin, it immediately precipitated at the bottom of the beaker. Thus, these samples were lumpy, and no spherical particles were formed. For **CMCG_7.5,0.4_**, although spherical particles were formed by floating in paraffin, a portion of the aqueous solution precipitated at the bottom, resulting in a relatively low yield (58%). For **CMCG_10,0.4_**, most of the reaction mixture was converted into spherical particles because of the increase in CMC concentration. Thus, the yield reached 90%, which was almost in agreement with the yield of the CMCG samples (80–91%) prepared without liquid paraffin [10]. However, when the CMC concentration was increased to 15 wt% (**CMCG_15,0.4_**), the aqueous solution adhered to the surface of the stirring blade, owing to its high viscosity. Furthermore, increasing the CMC concentration to 15 wt% decreased the formation of spherical particles with a relatively low yield of 65%. The yield data suggest that the concentration of CMC, which is related to the specific gravity and viscosity of the reaction solution, was critical for the formation of the spherical particles and that 10 wt% of the initial CMC concentration was optimal.

**Figure 2:**
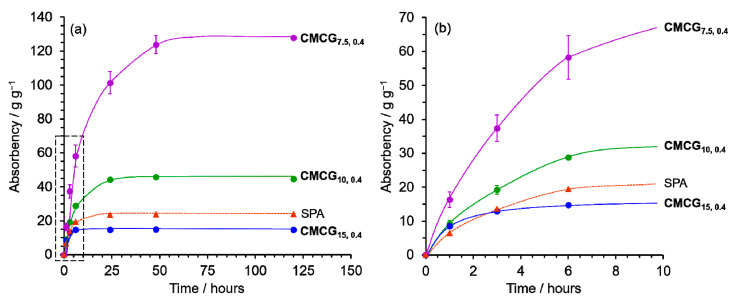
Time-dependence of the absorbency of the CMCGs and sodium polyacrylate (SPA) (**a**) toward phosphate-buffered saline (PBS) and (**b**) initial stage of the absorbency.

Then, the absorbency of **CMCG_7.5,0.4_**, **CMCG_5,0.4_**, and **CMCG_15,0.4_** was investigated using phosphate-buffered saline (PBS) as the absorbing solution. Figure 2 compares the time-dependency of the PBS absorbency for the CMCG samples and SPA. The absorbency increased with time and reached equilibrium after 24 h for **CMCG_10,0.4_**, **CMCG_15,0.4_**, and SPA, and after 48 h for **CMCG_7.5,0.4_**. The maximum absorbency at equilibrium increased with a decrease in the initial CMC concentration. This phenomenon can be attributed to the molecular structure of CMCG, including its degree of crosslinking. It has been reported that in a CMC hydrogel using epichlorohydrin, an epoxide crosslinking agent [24], the low concentration of CMC in the reaction mixture led to a decrease in the crosslinking density of the CMC chains. The Fourier transform infrared (FTIR) spectra of CMCGs suggest that the degree of crosslinking decreases with decreasing initial CMC concentration (Figure S2 and Table S1). The less crosslinked structure permits the expansion of the CMC molecules in the hydrogels, resulting in higher absorption.

**Figure 3:**
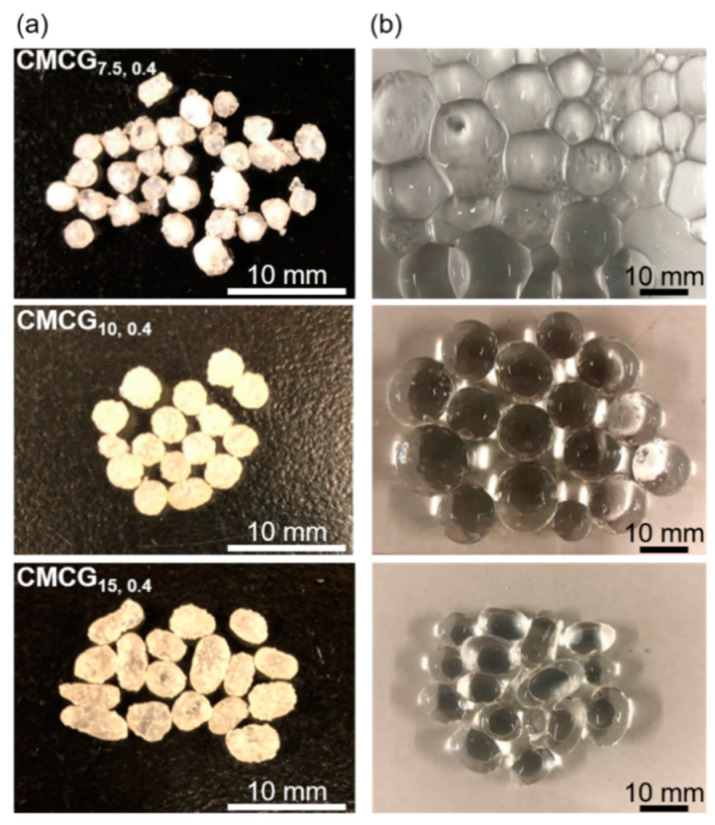
Photographs showing the appearance of the CMCGs: (**a**) dried and (**b**) swollen with PBS.

**Figure 4:**
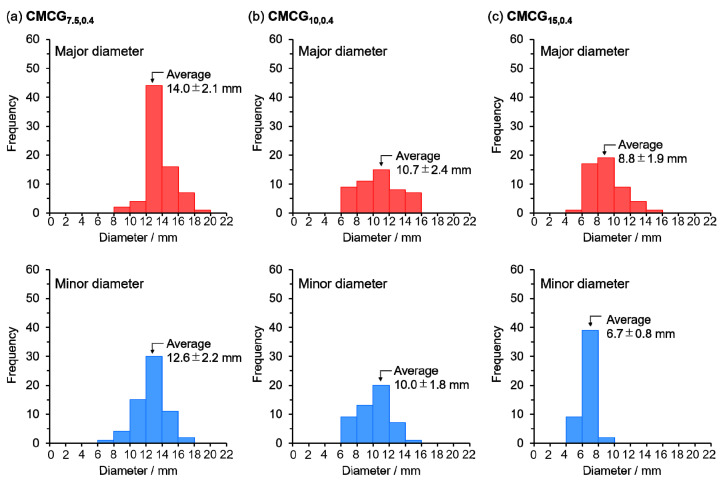
Size distributions of the major and minor diameters of swollen in PBS.

The photographs of **CMCG_7.5,0.4_**, **CMCG_5,0.4_**, and **CMCG_15,0.4_** in the dried and swollen states are shown in Figure 3, whereas Figure S4 shows photographs of SPA for comparison. The dried CMCGs were white or yellowish-white particles, which changed to more yellowish as the initial CMC concentration increased. Because EGDE is a slightly yellow-colored liquid and CMC is a white powder, this finding suggests that as the CMC concentration was increased, the crosslinking degree of EGDE also increased; this supports the absorbency results. The dried CMCG transformed into transparent spherical particles after swelling in PBS (Figure 3b). Furthermore, none of the CMCG samples deformed under their weight and retained their shape, except for **CMCG_7.5,0.4_**. This was because **CMCG_7.5,0.4_** had a low CMC concentration, which led to decreased crosslinking between the CMC chains. Because some of the CMCG particles were flattened, the major and minor diameters were measured from 50 random particles. Figure 4 shows the size distribution of the major and minor diameters of CMCG swollen with PBS. The average values of the major diameters were 14.0 ± 2.1 (**CMCG_7.5,0.4_**), 10.7 ± 2.4 (**CMCG_10,0.4_**), and 8.8 ± 1.9 mm (**CMCG_15,0.4_**). The average values of the minor diameters were 12.6 ± 2.2 (**CMCG_7.5,0.4_**), 10.0 ± 1.8 (**CMCG_10,0.4_**), and 6.7 ± 0.8 mm (**CMCG_15,0.4_**). The major and minor diameters decreased with increasing CMC concentrations. When the CMC solution was poured into the liquid paraffin, the CMC solution was separated, owing to the difference in surface tension between the water and liquid paraffin. The CMC solution was sliced by the shear force of the propeller blade and formed sphere droplets to reduce the surface area. However, the CMC solution was not only divided into droplets by the shear force but also coalesced into bigger droplets. As the reaction progressed, an intermolecular crosslinked structure was formed, and the CMC droplets were stably dispersed in the liquid paraffin without coalescing. A high CMC concentration is expected to increase the chance of contact between the unsubstituted hydroxy groups of CMC and EGDE, which results in the rapid formation of an intermolecular crosslinked structure. Thus, it is likely that an intermolecular crosslinked structure was formed in the early stages of the reaction, which stabilized the droplet shape. Furthermore, the size of the CMCG decreased as coalescence was suppressed. In contrast, at low CMC concentrations, the reaction progressed slower than at high CMC concentrations. Because it takes time to stabilize the droplet shape by intermolecular crosslink formation, the CMCG size increased, owing to the coalescing droplets. Furthermore, the high surface tension of the CMC solution led to a decrease in the droplet size. The surface tension increased with increasing solution viscosity, which increased with increasing CMC concentration. That is, the surface tension increased with increasing CMC concentration, which caused a decrease in the droplet size.

A ratio of the major to minor diameters close to 1 (i.e., the smaller the difference between the major and minor diameters) indicates that the particles are spherical. To provide a benchmark, the SPA had an average value of the major diameter of 10.6 ± 0.4 mm and a minor diameter of 10.5 ± 0.3 mm, resulting in a ratio of 1.00, indicating that the particles were spherical. CMCG, on the other hand, had ratios of 1.11 (**CMCG_7.5,0.4_**), 1.07 (**CMCG_10,0.4_**), and 1.31 (**CMCG_15,0.4_**). The ratio of **CMCG_10,0.4_** was closest to 1, indicating that the particles were almost spherical. **CMCG_15,0.4_** was obtained as non-spherical particles; the oval particles are apparent in the photographs (Figure 3b). The high viscosity of the CMC solution seems to have caused the miniaturization of the CMC solution while stretching and forming oval droplets by shear force. These results confirm that a CMC concentration of 10 wt% is appropriate for obtaining spherical CMCG and was used for the subsequent experiments.

#### 2.1.2. Effect of the Feed Amount of the Crosslinking Agent

**Figure 5:**
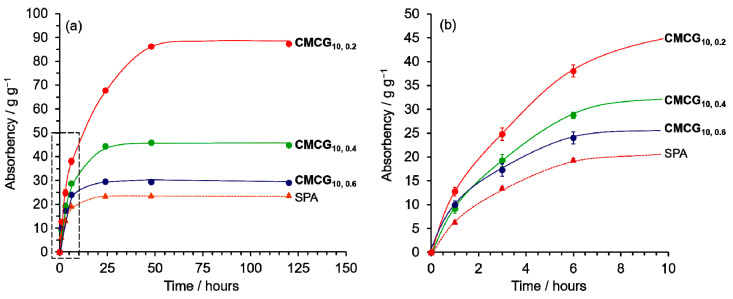
Time-dependence of the absorbency of the CMCGs and SPA (**a**) toward PBS and (**b**) initial stage of the absorbency.

The effect of the EGDE concentration (0.2, 0.4, and 0.6 g per 1 g of CMC; CMC concentration was kept constant at the optimal 10 wt%) on the CMCGs (**CMCG_10,0.2_**, **CMCG_10,0.4_**, and **CMCG_10,0.6_**) was determined. Figure 5 shows the time-dependency of the PBS absorbency with the CMCGs and that with SPA as a reference (dotted line). The absorbency gradually increased over time and reached equilibrium at its maximum after 24 h for **CMCG_10,0.4_**, **CMCG_10,0.6_**, and SPA, and after 48 h for **CMCG_10,0.2_**. The maximum absorbency of all the CMCGs at equilibrium was higher than that of SPA. These results suggest that the maximum absorbency at equilibrium decreases with an increasing EGDE/CMC ratio. In our previous study [10,25], the degree of crosslinking increased with an increase in the feed ratio of the crosslinking agent to CMC, during hydrogel preparation. According to the FTIR spectra of CMCGs, it is suggested that the degree of crosslinking of CMCG also increases as the amount of EGDE increases (Figure S3 and Table S1). The highly crosslinked structure suppressed the expansion of the CMC molecules in the hydrogels, resulting in lower absorption.

**Figure 6:**
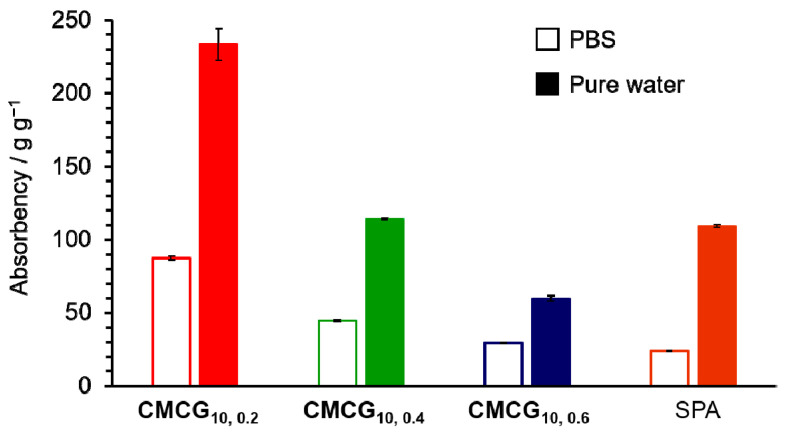
Absorbency of the CMCGs and SPA toward PBS (open column) and pure water (closed column) after 48 h.

Figure 6 shows the absorbencies of CMCG in PBS and pure water after 48 h. Both CMCG and SPA exhibited an absorbency toward pure water that was more than twice as high as that of PBS. In general, the absorbency of ionic gels depends on the difference in the cation concentration between the inner and outer liquids of the gels [26,27,28]. Ionic gels have functional groups (e.g., sodium carboxylates) that dissociate in water. The free cation, such as Na^+^, produced as a result, causes a concentration difference between the inner and outer solutions, and water permeates the gel by osmotic pressure. The difference in the ionic concentration between the inside of the gel and the outer solution decreases in electrolyte solutions, such as PBS. Thus, the absorbency toward PBS decreased because the water penetration was suppressed. The absorbency of SPA toward pure water was 109 g g^−1^, whereas that for PBS was approximately 20% of the water absorbency. In contrast, the absorbency of CMCG toward PBS was approximately 40–48% of that toward pure water. These values were higher than those of SPA. The CMC backbone contains several hydrophilic hydroxy groups. The unsubstituted hydroxy groups on the CMC backbone increased the affinity for water as hydrophilic groups, resulting in a higher absorbency toward PBS compared to SPA.

**Figure 7:**
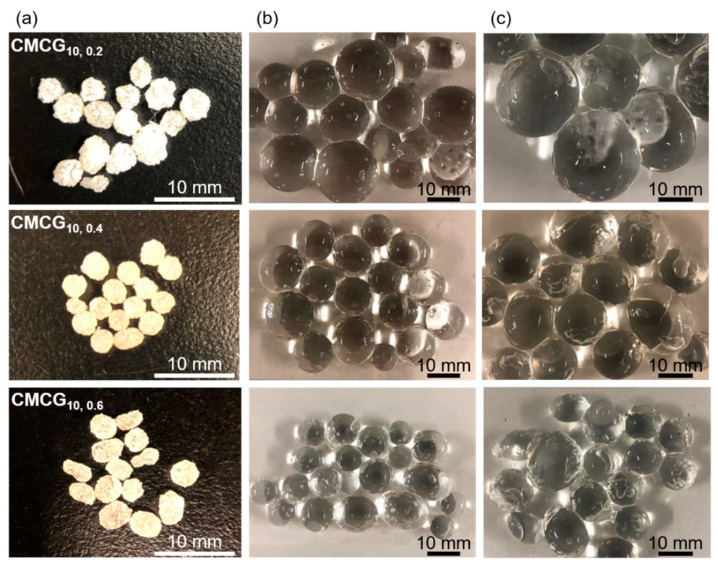
Photographs showing the appearance of the CMCGs: (**a**) dried, swollen with (**b**) PBS, and (**c**) pure water, after 48 h.

**Figure 8:**
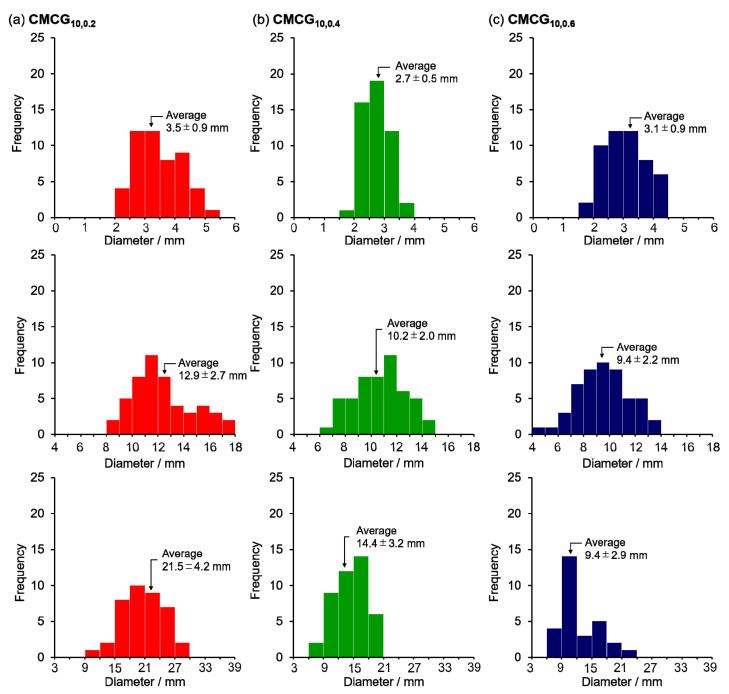
Size distributions of the CMCGs: dried (top) and swollen with PBS (middle) and pure water (bottom).

**Figure 9:**
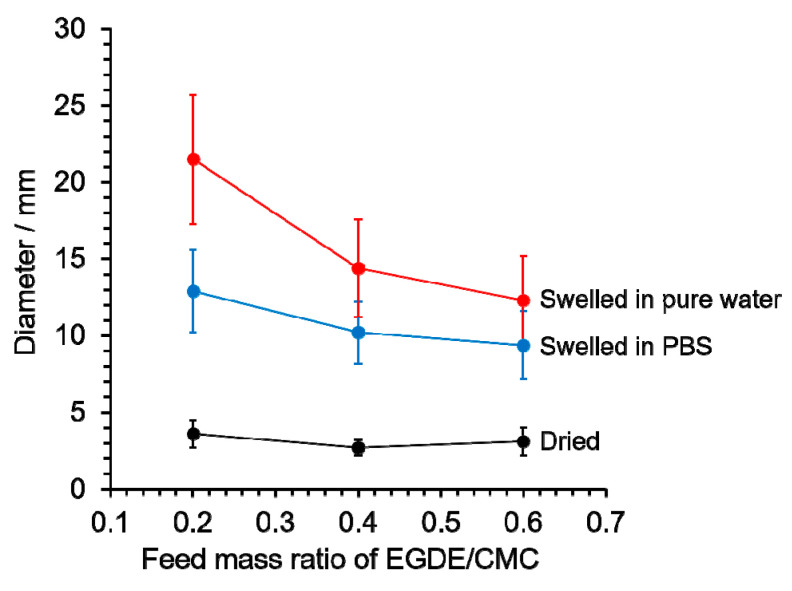
Effect of the mass ratio of ethylene glycol diglycidyl ether (EGDE)/cellulose sodium salt (CMC) on the dried CMCGs and on those swollen in PBS and pure water.

The photographs of CMCG before and after absorption in PBS and pure water are shown in Figure 7. The dried CMCG became a transparent spherical hydrogel after swelling in PBS. None of the CMCGs deformed under their weight and were able to retain their shape. Although some CMCG particles were flattened, nearly spherical particles were observed, indicating that the amount of EGDE feed did not affect the shape of CMCG. Following that, the major and minor diameters of each CMCG were measured, and the average value was used as the CMCGs’ diameter. The size distribution histograms of the CMCGs were obtained by measuring the diameters of 50 randomly selected CMCGs (Figure 8). The mean value and standard deviation were obtained from the histograms and are shown in Figure 9. The average diameter of the swollen CMCG decreased with an increasing EGDE feed amount. Similarly, the absorbency decreased, indicating that the increase in crosslinking density resulted in the construction of a strong network structure, which suppressed the expansion of the network and decreased the size of the swollen particles. The absorbency and size of the CMCG could be controlled by the feed amount of EGDE. So far, the results reveal that the CMC concentration mostly determined the shape of CMCG, whereas the feed amount of EGDE controlled its absorbency.

Because the absorbency of water was higher than that of PBS, the particle size of CMCG that swelled toward pure water was larger than that of PBS. The intermolecular network structure expanded because of the large amount of water absorbed, and the CMCG was brittle and exhibited cracks. The higher the mass ratio of EGDE/CMC, the more cracks were observed in the CMCG. The degree of crosslinking increased with an increase in the mass ratio of EGDE/CMC, and the flexibility between the molecular chains decreased. The cracks were caused by the inclusion of a large amount of water between the molecular chains with reduced flexibility, owing to the high degree of crosslinking.

The absorbency in PBS of the CMC hydrogel crosslinked with polyethylene glycol diglycidyl ether (PEGDE) as a crosslinking agent was 90–230 g·g^−1^ [25], which is higher than that of CMCG (30–90 g·g^−1^). The absorbency of a CMC hydrogel crosslinked with PEGDE is similarly affected by the PEGDE feed quantity, which is 0.04–0.23 equivalents per CMC anhydroglucose unit. In contrast, CMCGs were prepared by adding 0.25–0.75 equivalents of EGDE per anhydroglucose unit of CMC, which was an excess of PEGDE. By reducing the amount of EGDE added, it is envisaged that CMCG will be able to reproduce the same level of absorption as the CMC hydrogel with PEGDE. However, as observed in the images of CMCG swollen with pure water, the spherical shape may not be maintained, due to cracks induced by the significant amount of absorption (Figure 7).

### 2.2. Water-Holding Capacity

**Figure 10:**
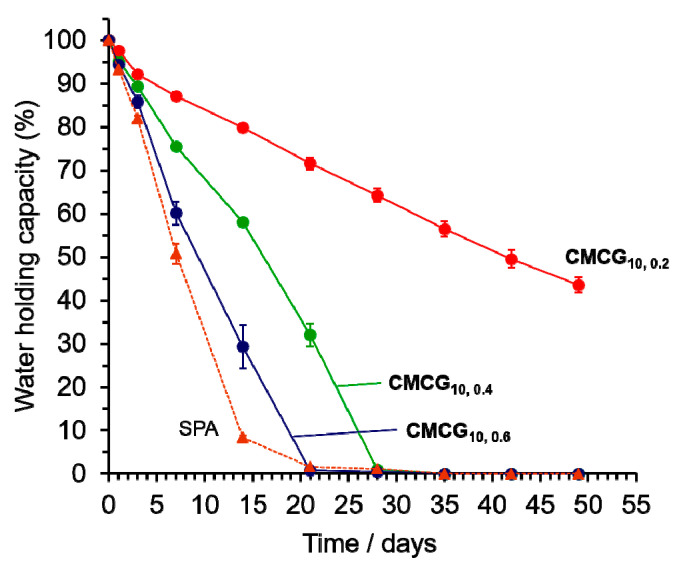
Time-dependence of the water-holding capacities of the CMCGs and SPA at a temperature of 298 K and a humidity of 55%.

After testing for absorbency, the water-holding capacity of the CMCG swollen with PBS was determined by placing it in a chamber at a temperature of 298 K and a humidity of 55%. The time-dependence of the water released from the CMCG is shown in Figure 10. The water-holding capacity of the SPA is presented as a dotted line in the figure for comparison. All the hydrogels, including SPA, released water over time. On day 1 of the test, the water-holding capacity for all the hydrogels was almost the same. However, that of SPA rapidly decreased after 3 days and was 14.8% after 14 days. The water-holding capacities of the CMCGs also gradually decreased with time, but were 80% (**CMCG_10,0.2_**), 58% (**CMCG_10,0.4_**), and 30% (**CMCG_10,0.6_**) on day 14, which were higher than those of SPA. In the CMCGs, the water release rate was inhibited, suggesting that the unsubstituted hydroxy groups of cellulose increased its affinity toward water. However, the release rate of water accelerated with an increase in the feed amount of EGDE to CMC. The water-holding capacity was almost 0% after 21 days for **CMCG_10,0.6_**, and 28 days for **CMCG_10,0.4_**. In contrast, **CMCG_10,0.2_** showed a high water-holding capacity of 42% even after 49 days. This was because the degree of crosslinking increased, potentially leading to a decrease in the number of unsubstituted hydroxy groups as the mass ratio of EGDE/CMC increased. It is suggested that a decrease in the number of hydroxy groups decreases the affinity toward water and causes an increase in the rate of water release.

### 2.3. Cellulase Degradability

**Figure 11:**
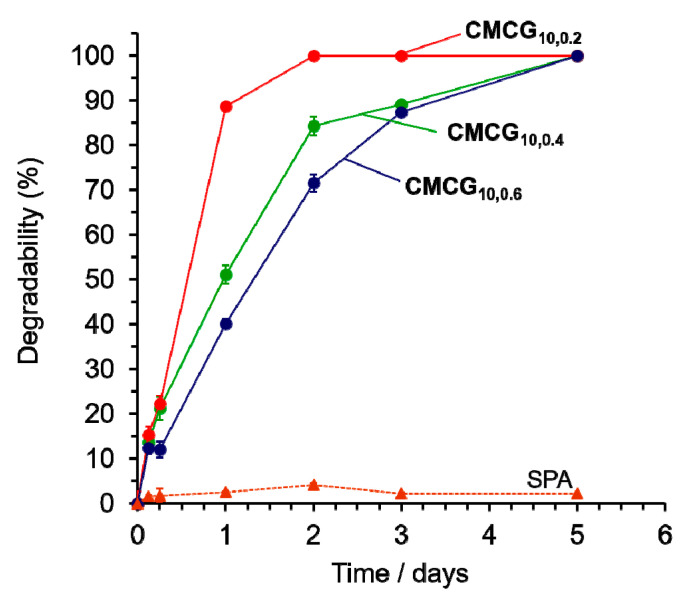
Time-dependency of the degradability of the CMCGs and SPA by cellulase in the 100 mM sodium acetate buffer (pH 5.0) at 313 K.

CMCGs with different feed mass ratios of EGDE/CMC were enzymatically degraded using cellulase in a nylon teabag. The degradation was conducted using cellulase derived from *Trichoderma*, which is commonly found in soil [29]. The cellulase degraded the CMCG into low molecular weight fractions, which were eluted from the nylon teabag. Cellulase degradability was calculated from the difference between the weight of the CMCG remaining in the teabag and its initial weight. The time-dependence of degradation is shown in Figure 11. The dotted line in this figure represents the weight change in the SPA, which is almost unchanged over time, indicating that SPA was not degraded by cellulase because it is a polyacrylic acid. In contrast, the cellulase degradability of all the CMCG samples was excellent and increased with time. After 7 days, all the CMCGs were completely degraded. **CMCG_10,0.2_**, exhibited 89% degradation in 1 day, which confirmed that CMCG was almost completely degraded after 2 days. **CMCG_10,0.6_** showed degradation of 40% in 1 day and required 5 days to be completely degraded. That is, the degradation rate decreased with an increase in the mass ratio of EGDE/CMC. The degradation of cellulose-based hydrogels is not dependent on the water absorbency and water-holding capacity, but decreases because the interior or surface cellulose chains are not easily accessible to cellulase with an increasing degree of crosslinking [10,25,30,31]. This suggests that the contact between cellulase and the cellulose chains of CMCG was inhibited as the mass ratio of EGDE/CMC increased, which caused a decrease in degradation in the initial stages. However, as the degradation of CMCG progressed, cellulase was able to access the exposed cellulose chains, leading to the complete degradation of CMCG. Furthermore, the cellulase degradability of CMC hydrogels crosslinked with EGDE and PEGDE was a maximum of 70%/5 days and 62%/3 days, depending on the feed amount of crosslinking agent [10,25]. This degradability was calculated based on the amount of the reducing sugars released by the cellulase degradation, indicating complete degradation of CMC to glucose. The degradability of CMCG was determined in this study based on the change in mass of the CMCG remaining in the teabag before and after cellulase degradation, and the low molecular weight fractions eluted from the teabag are also included in the degradability. Therefore, it is expected that CMCG will take longer to completely degrade to glucose than the day it exhibited 100% degradability. Although more research into the microbial degradation of CMCG in the natural environment, such as soil, is required, these findings indicate that CMCG can be degraded by microorganisms.

## 3. Conclusions

The crosslinking reaction of CMC with EGDE was conducted in an aqueous droplet of liquid paraffin, formed by the shear force from stirring and the surface tension of the water–oil interface, yielding spherical hydrogel particles. The particle size decreased as the CMC concentration increased, and the shape turned non-spherical. A CMC concentration of 10 wt% was optimal to obtain spherical CMCG. Furthermore, increasing the mass ratio of EGDE/CMC suppressed the electrostatic repulsion between the CMC molecules in the gel structure, decreasing the absorbency. These findings suggest that particle shape and absorbency can be controlled by CMC concentration and EGDE feed quantity, respectively. The spherical CMC hydrogel particles were completely degraded by cellulase. A strong dependence of the degradation rate on the feed amount of EGDE was observed, where the degradation rate increased with a decrease in the feed amount of EGDE. Our results indicate that the spherical hydrogel particles prepared in this study have the potential for a variety of uses, such as air fresheners and horticultural water-holding agents, as alternatives to current synthetic SAPs. In addition, since the spherical CMCG has numerous carboxy groups in its structure, further functionalization can be expected by utilizing them as cross-linking points [32,33]. Nonetheless, the raw material cost of CMCG is higher than the price of existing synthetic SAP, and further research, including cost reduction, is required to establish its utility as an SAP alternative.

## 4. Materials and Methods

### 4.1. Materials

CMC (molecular weight = 8–10 × 10^4^ as specified by manufacturer) with a 0.70 degree of substitution was obtained from Daicel Co., (Osaka, Japan). EGDE was purchased from Fujifilm Wako Pure Chemical Co., Ltd. (Osaka, Japan). Liquid paraffin (Moresco white P-100, 19.04 mm^2^/s) was purchased from Moresco Co., (Kobe, Japan). Sodium hydroxide was purchased from Kanto Chemical Co., Inc. (Tokyo, Japan). Methanol was purchased from Godo Co., Ltd. (Tokyo, Japan). The SPA was supplied by S. T. Co., (Tokyo, Japan). *Trichoderma viride* cellulase ONOZUKA R-10 was purchased from Yakult Pharmaceutical Co., Ltd. (Tokyo, Japan). The PBS and sodium acetate buffer used were analytical grade chemicals, i.e., sodium chloride, potassium chloride, sodium dihydrogen phosphate, potassium dihydrogen phosphate, sodium acetate, and acetic acid, purchased from Kanto Chemical Co., Inc. (Tokyo, Japan).

### 4.2. Preparation of CMCG

First, CMC (1.0 g) was completely dissolved in 0.5 mol L^−1^ aqueous NaOH (40 mL), followed by adding EGDE (0.40 g) with stirring at 250 rpm for 20 min using a Teflon impeller. Then, the reaction mixture was injected into 200 mL of liquid paraffin in a 300 mL beaker while stirring at 300 rpm, using stainless steel propeller blades at 328 K for 3 h to allow the crosslinking reaction of CMC to occur. Once the reaction was complete, the CMCG was washed with deionized water containing 1 wt% natural detergent, until the liquid paraffin on the surface of the CMCG was completely removed. Subsequently, the CMCG was immersed in methanol until pH 7.0 was reached to remove any unreacted materials and NaOH. Finally, it was dried at 318 K under reduced pressure (CMCG_2.5,0.4_). The dried CMCG samples larger than 2 mm were collected using a sieve with a 2 mm mesh opening, which were used in various tests. The CMCG samples with varying CMC and EGDE concentrations were prepared using a similar method (Table 1).

### 4.3. Water Absorbency

The absorbency of CMCG in PBS was determined using the following method: dried CMCG (250 mg) was immersed in a PBS solution at 298 K. After 1, 3, 6, 24, 48, and 120 h, CMCG was removed from the PBS solution, and any excess solution was drained onto a mesh for 5 min. The weight of the hydrogels was measured and the absorbency was calculated using Equation (1), which is as follows:

(1) $$\mathrm{Absorbency}=\frac{W_{s}-W_{d}}{W_{d}},$$

where *W_d_* and *W_s_* are the weights of the dried and swollen CMCG, respectively, at a certain time. The absorbency measurements were performed three times. The absorbency of CMCG in pure water instead of PBS was also similarly investigated. For comparison, the same experiments were performed using the SPA instead of CMCG.

### 4.4. Determination of Particle Size

Photographs of the dried and swollen CMCG were taken, and the digitalized images were analyzed using the *Image J* (version 1.53 m) image analysis program developed by the U.S. National Institutes of Health. Because the particles were flattened shapes, as observed in the photographs, the major and minor diameters were measured using dried and swollen CMCG. The size distribution was determined with the diameters calculated using 50 randomly selected CMCG in the photographs. Using data obtained from 50 CMCGs, the mean diameters and standard deviations were calculated.

### 4.5. Water-Holding Capacity

The CMCG (250 mg) immersed in PBS solution at 298 K for 5 days was placed in a chamber maintained at a temperature and humidity of 298 K and 55%, respectively. After 1, 3, 7, 14, 21, 28, 35, 42, and 49 days, the weight of the CMCG was measured. The water-holding capacity was determined using Equation (2), which is as follows:

(2) $$\mathrm{Water}\;\mathrm{holding}\;\mathrm{capacity}=\frac{W_{p}-W_{d}}{W_{i}-W_{d}}\times 100$$

where *W_i_* is the weight of the swollen CMCG at its initial state and *W_p_* is the weight of the swollen hydrogel at a certain time. *W_d_* is the initial weight of the dried CMCG. The water-holding capacity was measured thrice. For comparison, the same experiments were performed using SPA.

### 4.6. Cellulase Degradation

Cellulase degradability was determined based on the change in the CMCG weight before and after the enzymatic reaction. A teabag (50 mm × 50 mm) was prepared from a nylon sheet with a pore size of 255 mesh using a heat sealer. Then, CMCG (200 mg) was placed in the teabag, which was immersed in 29 mL of 100 mM sodium acetate buffer (pH 5.0) for 1 day. Cellulase dissolved in 1 mL of the same buffer solution was added to the solution containing CMCG to give a cellulase concentration of 5 × 10^−5^ wt%. The teabag immersed in the solution was incubated at 313 K with shaking at 100 rpm. After 3, 6 h, and 1, 2, 3, 5 days, the teabag was removed from the solution and left in a boiling bath for 10 min to inactivate the cellulase. Thereafter, the teabag was dried in an oven at 378 K, and the weight of the teabag (*W_a_*) was measured. Cellulase degradability was calculated using Equation (3), which is as follows:

(3) $$\mathrm{Degradability}=\frac{W_{d}-(W_{a}-W_{t})}{W_{d}}\times 100$$

where *W_d_* is the initial weight of the dried CMCG and *W_t_* is the weight of the empty teabag. Cellulase degradation was performed in triplicate. For comparison, the same experiments were performed using SPA.

## Figures and Tables

**Table 1:** Initial feed amount of carboxymethyl cellulose sodium salt (CMC) and ethylene glycol diglycidyl ether (EGDE) and yields from the preparation of the spherical CMC hydrogel (CMCG) samples.

Sample ^a^	Initial Feed Amount		CMC Concentration (wt%) ^c^	Feed Mass Ratio of EGDE/CMC	Yields ^d^
	CMC (AGU ^b^)	EGDE			
**CMCG_2.5,0.4_**	1.0 g (4.6 mmol)	0.4 g (2.3 mmol)	2.5	0.4	-
**CMCG_5,0.4_**	2.0 g (9.2 mmol)	0.8 g (4.6 mmol)	5	0.4	-
**CMCG_7.5,0.4_**	3.0 g (13.8 mmol)	1.2 g (6.9 mmol)	7.5	0.4	2.5 g (58%)
**CMCG_10,0.4_**	4.0 g (18.3 mmol)	1.6 g (9.2 mmol)	10	0.4	5.3 g (90%)
**CMCG_15,0.4_**	6.0 g (27.5 mmol)	2.4 g (13.8 mmol)	15	0.4	5.6 g (65%)
**CMCG_10,0.2_**	4.0 g (18.3 mmol)	0.8 g (4.6 mmol)	10	0.2	4.3 g (86%)
**CMCG_10,0.6_**	4.0 g (18.3 mmol)	2.4 g (13.8 mmol)	10	0.6	4.0 g (60%)

^a^ X and Y in the abbreviation CMCG_X,Y_ refer to the CMC concentration (wt%) and mass ratio of EGDE/CMC, respectively. ^b^ AGU is the mole of anhydroglucose unit of CMC. ^c^ CMC dissolved in 40 mL of 0.5 M NaOH. ^d^ Yields (%) were determined using the following equation: (mass of CMCG/g) × 100/(sum of mass of CMC and EGDE/g).

## Data Availability

The data presented in this study, supporting the results, are available in the main text. Additional data are available upon request from the corresponding authors.

## Supplementary Materials

The following supporting information can be downloaded at: https://www.mdpi.com/article/10.3390/gels8050321/s1, Figure S1: Photographs showing the appearance of the CMCGs swollen with PBS after 48 h. CMCG was prepared at stirring speeds of (a) 300 rpm and (b) 400 rpm; Figure S2: FTIR spectra of CMCGs with different CMC concentrations and CMC as a reference. These spectra were normalized by the peak intensity of the carboxylate groups at 1593 cm^−1^; Figure S3: FTIR spectra of CMCGs with different feed ratios of EGDE/CMC and CMC as a reference. These spectra were normalized by the peak intensity of the carboxylate groups at 1593 cm^−1^; Table S1: Peak area ratio of methylene group to carboxylate group (*A_CH2_/A_COONa_*) from FTIR spectra; Figure S4: Photographs showing the appearance of SPA: (a) dried, swollen with (b) PBS and (c) pure water, after 48 h.
