# Supplementary material for: Preparation and Enzyme Degradability of Spherical and Water-Absorbent Gels from Sodium Carboxymethyl Cellulose
Source: Gels. 2022 May 20;8(5):321. doi: 10.3390/gels8050321 (PMC9141264; doi:10.3390/gels8050321)
Supplement: Supplementary file 1 [file gels-08-00321-s001.zip › gels-1664272-supplementary.pdf]

gels

Article

Preparation and Enzyme Degradability of Spherical and Water-Absorbent Gels from Sodium Carboxymethyl Cellulose

Sayaka Fujita [1,*], Toshiaki Tazawa [2] and Hiroyuki Kono [1,*]

1 Division of Applied chemistry and Biochemistry, National Institute of Technology, Tomakomai College, Tomakomai 059-1275, Japan
2 R&D Center, S.T. Corporation, Shinjuku-ku, Tokyo 161-0033, Japan; t-tazawa@st-c.co.jp
* Correspondence: fujita@tomakomai-ct.ac.jp (S.F.); kono@tomakomai-ct.ac.jp (H.K.); Tel.: +81-144-67-8038 (S.F.); +81-144-67-8036 (H.K.)

Supporting Information

Effect of the stirring speed on the CMCG particle sizes

The effect of stirring speed on the particle size was investigated. CMCG were synthesized from CMC (degree of substitution 0.80, molecular weight = 3–4 × 10^5 as specified by the manufacturer, CP Kelco Co., Ltd. (Atlanta, GA, USA)) using the method described in section 4.2. The CMC concentration and the feed mass ratio of EGDE/CMC were set to 10 wt.% and 0.4, respectively, and the stirring speed was set at either 200, 300 or 400 rpm. At a stirring speed of 200 rpm, the CMC mixture accumulated at the bottom of the beaker, and no particles were obtained. At over 300 rpm, the particles were obtained, and the photographs of particles swollen with PBS are shown in Figure S1.

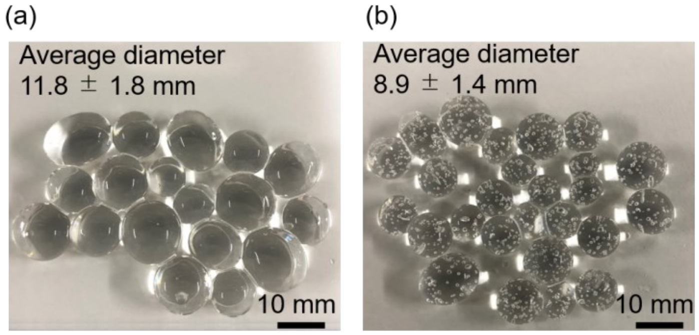

Figure S1. Photographs showing the appearance of the CMCGs swollen with PBS after 48 h. CMCG was prepared at stirring speeds of (**a**) 300 rpm and (**b**) 400 rpm.

Measuring of Fourier transform infrared spectroscopy (FTIR) of CMCG

The FTIR spectra were recorded on a Spectrum II FTIR spectrometer (PerkinElmer, Inc., Waltham, MA, USA). FTIR spectra were recorded after grinding the sample into a powder and mixing well with potassium bromide powder. The powder mixture was compressed into a transparent disk and scanned from 4000 to 500 cm^{-1} using an average of 16 scans, with a resolution of 1 cm^{-1}. After the baseline correction, the peak areas were integrated utilizing the PerkinElmer Spectrum 10 software package (version 10.4.3, PerkinElmer, Inc., Waltham, MA, USA).

Figures S2 and S3 show the FTIR spectra of CMCGs and the starting material of CMC. These spectra display common absorption bands at 2943, 2896, and 1593 cm^{-1} that can be assigned to asymmetric stretching vibration of the aliphatic CH_2, symmetric stretching vibration of CH_2, asymmetric vibration of carboxylate groups, respectively. Since the carboxylate groups in the CMC are unchanged by the crosslinking reaction with EGDE for

the preparation of CMCG samples, these spectra were normalized by the absorption of the carboxylate group at 1593 cm^{-1}.

In order to quantitatively evaluate the number of EGDE molecules crosslinked to CMC chains in the CMCGs, integration values for absorption of CH_2 (A_{CH2}, 2990–2700 cm^{-1}) and that of carboxylates (A_{COONa}, 1820–1510 cm^{-1}) were determined (Figures S2 and S3). The integral area ratios (A_{CH2}/A_{COONa}) for CMCGs and CMC are summarized in Table S1. The A_{CH2}/A_{COONa} of CMC is 0.22, whereas those of **CMCG$_{7.5, 0.4}$**, **CMCG$_{10, 0.4}$**, and **CMCG$_{15, 0.4}$** are 0.27, 0.28, and 0.33, respectively. This gradual increase of A_{CH2}/A_{COONa} indicates that crosslinking efficiency by EGDE is enhanced with the increase of CMC concentration for the sample preparation, which results in the increase of crosslinking degree of CMCG [10]. Similarly, it was suggested that an increase in the feed amount of EGDE enhanced the crosslinking degree of CMCG from the finding that the A_{CH2}/A_{COONa} of **CMCG$_{10, 0.2}$**, **CMCG$_{10, 0.4}$**, and **CMCG$_{10, 0.6}$** are 0.24, 0.28, and 0.35, respectively.

In the previous research on the structural characterization of CMC-based hydrogels prepared by the crosslinking reaction of CMC using epichlorohydrin [10] or PEGDE [25] as a crosslinking agent, it was revealed that the absorption intensity of CH_2 at 2896 cm^{-1} normalized by the that of carboxylate group at 1593 cm^{-1} strongly correlates with the crosslinking degree determined by the solid-state NMR. Therefore, it is considered that solid-state NMR analysis of the CMCG samples is effective in discussing the molecular structure as well as molecular dynamics of these samples in detail.

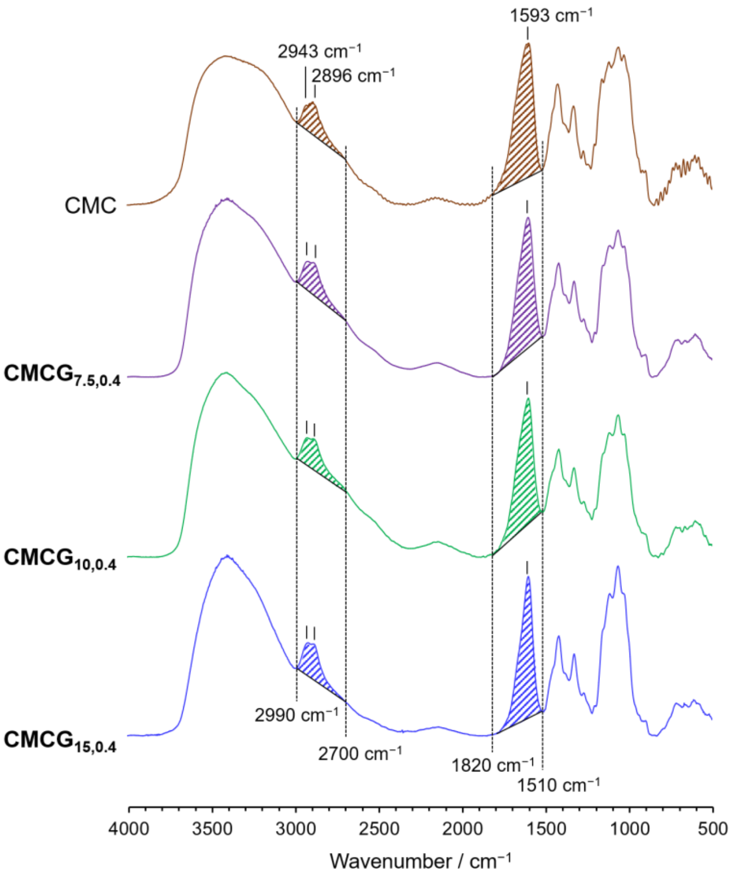

Figure S2. FTIR spectra of CMCGs with different CMC concentrations and CMC as a reference. These spectra were normalized by the peak intensity of the carboxylate groups at 1593 cm^{-1}.

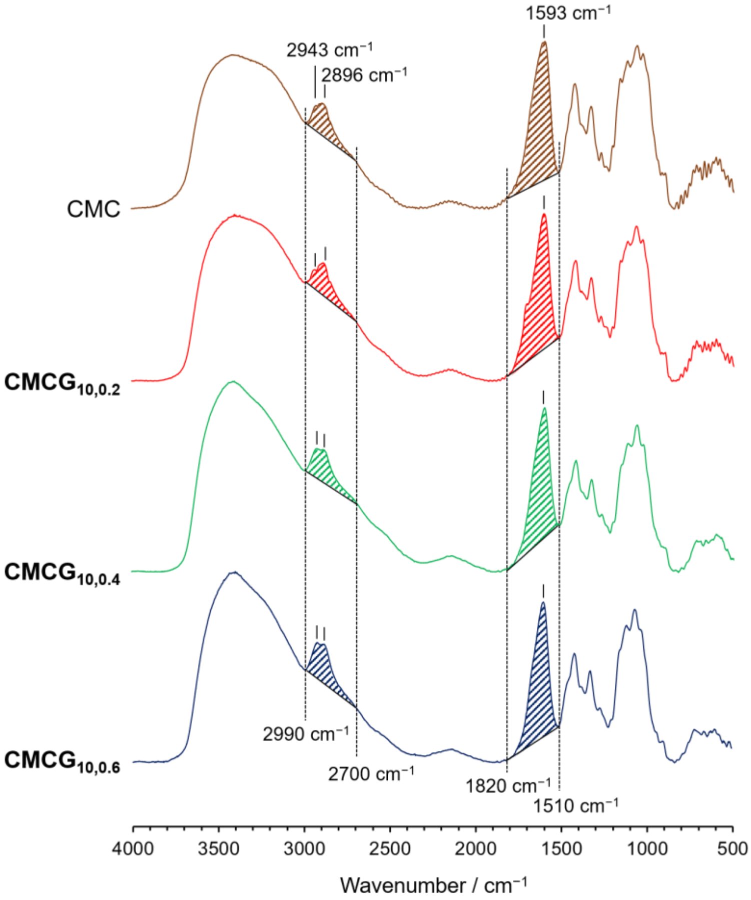

Figure S3. FTIR spectra of CMCGs with different feed ratios of EGDE/CMC and CMC as a reference. These spectra were normalized by the peak intensity of the carboxylate groups at 1593 cm^{-1}.

Table S1. Peak area ratio of methylene group to carboxylate group (A_{CH2}/A_{COONa}) from FTIR spectra.

Sample	A_{CH2}/A_{COONa}
**CMC**	0.22
**CMCG$_{7.5, 0.4}$**	0.27
**CMCG$_{10, 0.4}$**	0.28
**CMCG$_{15, 0.4}$**	0.33
**CMCG$_{10, 0.2}$**	0.24
**CMCG$_{10, 0.6}$**	0.35

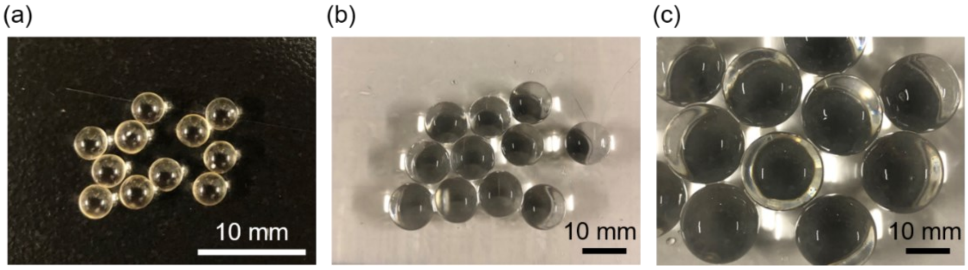

Figure S4. Photographs showing the appearance of SPA: (**a**) dried, swollen with (**b**) PBS and (**c**) pure water, after 48 h.
